# Leptospirosis, American Samoa

**DOI:** 10.3201/eid1812.120429

**Published:** 2012-12

**Authors:** Colleen L. Lau, John M. DePasquale

**Affiliations:** Author affiliations: The University of Queensland, Herston, Queensland, Australia (C.L. Lau);; Lyndon Baines Johnson Tropical Medical Center, Pago Pago, American Samoa (J.M. DePasquale)

**Keywords:** leptospirosis, Weil disease, tropical medicine, communicable diseases, emerging infectious diseases, dengue, zoonoses, laboratory diagnosis, bacteria, American Samoa

**To the Editor:** Leptospirosis is common in the Pacific Islands (66.4 cases/100,000 population/year compared with 5 cases/100,000 population/year globally) (*1*) and is often misdiagnosed as dengue because of overlapping clinical features, poor awareness, and inadequate diagnostic facilities (*2*,*3*). Clinical manifestations range from asymptomatic to severe disease with pulmonary hemorrhage and renal and hepatic failure.

Global emergence of leptospirosis has been associated with environmental factors including rainfall, flooding, poverty, urbanization, and ecotourism (*1*–*4*), to which the Pacific Islands are vulnerable. Seroprevalence of leptospirosis in American Samoa is 15.5% (*5*), and recent reports confirm its emergence in the Pacific region (*6*). We report a case of severe leptospirosis in American Samoa (one of the world’s wettest inhabited places) and illustrate diagnostic challenges and the need to improve laboratory capacity.

In January 2011 (wet season), a 15-year-old previously healthy Polynesian boy was examined for a 3-day history of fever, myalgia, fatigue, headache, sore throat, pleuritic chest pain, and vomiting. He spent much time outdoors, occasionally slept in the rainforest, and had recently waded through water.

Examination revealed lethargy, injected conjunctivae, mild periumbilical tenderness, fever (38.6°C), tachycardia (133 beats/minute), and hypotension (96/50 mm Hg). Lung sounds were clear, respiratory rate was 22 breaths/minute, and oxygen saturation was 99%. No rash or jaundice was noted. Laboratory investigations showed leukocytosis (9.35 × 10^9^ cells/L), neutrophilia (85%), mild normocytic anemia (12.0 g/dL), and thrombocytopenia (42 ×10^9^ platelets/L); chest radiographs showed mild infiltrate in the left lung.

Differential diagnosis included dengue, influenza, pneumonia, and leptospirosis. The patient was hospitalized for supportive treatment, but the next day he experienced shoulder pain, increased abdominal and chest pain, worsened thrombocytopenia, hypokalemia, hyperbilirubinemia, proteinuria, hematuria, and fecal occult blood. No abnormalities were found for the following: transaminase, alkaline phosphatase, blood urea nitrogen, and creatinine levels; blood culture; serologic test results for hepatitis; and abdominal ultrasonogram.

Intravenous penicillin was given for possible leptospirosis and/or pneumonia. Within 1 hour, the patient’s condition deteriorated: temperature increased (40.2°C); and rigors, severe headache, and myalgia developed. Jarisch-Herxheimer reaction was considered (*7*), and intravenous penicillin was replaced with ceftriaxone. The patient deteriorated further and exhibited hypotension, tachycardia, tachypnea, jaundice, confusion, mucosal bleeding, and required intensive care treatment, including intravenous dopamine for shock. Repeat chest radiograph showed deterioration with bilateral infiltrates. The Figure shows progression of kidney and liver function and thrombocytopenia.

**Figure:**
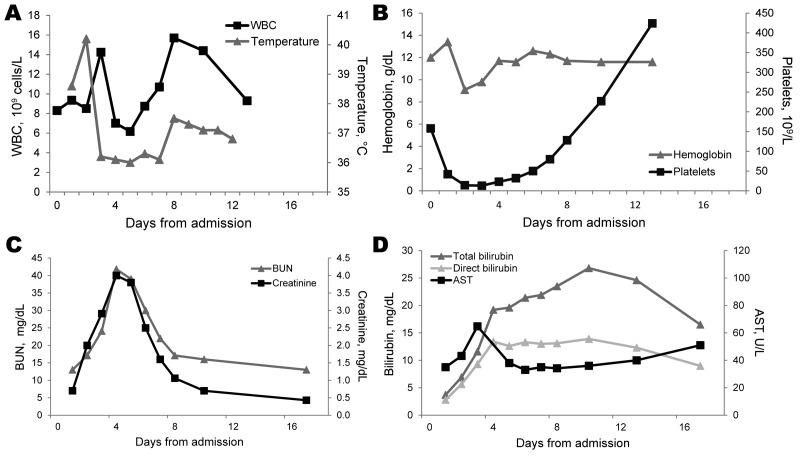
Clinical course of temperature and selected laboratory results in a patient with severe leptospirosis in American Samoa. A) Temperature and leukocyte (WBC) counts (reference range 5.0–9.1 × 10^9^ cells/L). B) Hemoglobin (reference range 14.0–16.3 g/dL) and platelet counts (reference range 150–450 × 10^9^ platelets/L). C) Blood urea nitrogen (BUN) (reference range 5–18 mg/dL) and creatinine (reference range 0.5–1.0 mg/dL) levels. D) Total bilirubin (reference range 0.3–1.2 mg/dL), direct bilirubin (reference range 0–0.2 mg/dL), and aspartate aminotransferase (AST) (reference range 15–45 U/L) levels.

Serum collected on hospitalization day 2 was negative for IgM against *Leptospira* spp. according to the GenBio IgM ImmunoDOT test (San Diego, CA, USA) (*8*). Serum collected on day 3 was positive for IgM and IgG against dengue virus according to the TECO rapid diagnostic test (Anaheim, CA, USA) (*8*), suggesting dengue hemorrhagic fever. The patient continued to receive intravenous ceftriaxone because of worsening condition and clinical suspicion of leptospirosis. He improved with antimicrobial drug treatment and supportive care, and his serum on day 9 was positive for IgM against *Leptospira* spp.

Subsequent serologic testing in Brisbane, Australia, confirmed leptospirosis and excluded dengue. Microscopic agglutination test results confirmed acute infection with *L. interrogans* serovar Copenhageni; rising titers were found in serum collected on days 3 (<50), 8 (100), and 17 (400). All samples were negative for IgM and IgG against dengue virus according to the PanBio Dengue IgM and IgG Capture ELISA tests (Sinnamon Park, Queensland, Australia) (sensitivity 99.2%, specificity 96.2%) (*9*).

This case illustrates that leptospirosis in the Pacific Islands presents many clinical challenges. This patient experienced a life-threatening illness with multiple complications associated with severe leptospirosis, including possible Jarisch-Herxheimer reaction. Early diagnosis is crucial because appropriate treatment with antimicrobial drugs can reduce illness and death (*1**,**2*). Molecular techniques provide rapid diagnosis during the bacteremic phase but are expensive and often unavailable in developing countries (*1**–**3*). Rapid tests for dengue virus have limited sensitivity and specificity and can produce false-positive results in patients with leptospirosis and other conditions (*8*). Serologic testing for leptospirosis detects acute infections only after the second week of illness, so it was crucial that leptospirosis was not excluded early when results were positive for dengue virus but negative for *Leptospira* spp.

During January 2009–June 2011, incidence rates for dengue and leptospirosis among children <16 years of age in American Samoa were 517 and 159 cases per 100,000 population per year, respectively; incidence was highest in the wettest months. Incidence of each infection peaked in October 2009 (1,512 and 798 cases/100,000 population/year), possibly related to the late September 2009 tsunami (*10*).

Flooding increases risk for dengue infection (by providing mosquito breeding sites) and leptospirosis (by disseminating leptospires in the environment and increasing human–animal contact). Concurrent outbreaks and co-infections are not uncommon and can complicate diagnosis. Incidence rates for both infections will probably increase with climate change in the Pacific region (*4*). Cocirculation of dengue serotypes increases incidence of dengue hemorrhagic fever and dengue shock syndrome, which are difficult to clinically distinguish from severe leptospirosis. Jarisch-Herxheimer reactions in leptospirosis patients treated with antimicrobial drugs can further complicate diagnosis (*7*). To reduce leptospirosis in the Pacific Islands, awareness of the disease, understanding of limitations of rapid diagnostic tests, and more regional laboratory capacity are needed.
